# A case of Ehlers–Danlos syndrome presenting with widened atrophic scars of forehead, elbow, knee, and pretibial area: A case report

**DOI:** 10.1097/MD.0000000000017138

**Published:** 2019-09-13

**Authors:** Xiangwen Xu, Zhichao Wang, Tao Zan

**Affiliations:** Department of Plastic and Reconstructive Surgery, Shanghai Ninth People's Hospital, Shanghai JiaoTong University School of Medicine, Shanghai, China.

**Keywords:** classical Ehlers–Danlos syndrome, COL5A2, mutation, whole exome sequencing

## Abstract

**Rational:**

Ehlers–Danlos syndrome (EDS) is a heritable connective tissue disorder. Currently, the genotype-phenotype correlations of classical EDS (cEDS) are still controversial. Hence, this study reported a case of cEDS with both clinical manifestations and COL5A2 gene mutation.

**Patient concerns:**

A 30-year-old female presented to the plastic-surgery clinic with widen atrophic scars on forehead, elbows, knees and pretibial area that had developed since childhood.

**Diagnosis:**

With the skin hyperextensibility, joint hypermobility, papyraceous scar revealed by physical examination, and the heterozygous pathogenic variant c1997G > A (p.P659P) in COL5A2 gene revealed by whole exome sequencing, the diagnosis of the classical Ehlers–Danlos syndrome was made.

**Interventions:**

The patient underwent facial scar resection and sutured in minimizing tension and perfect apposition to avoid the post-surgery scar formation.

**Outcomes:**

Follow-up 6 months after surgery, the wound remained a fine line scar.

**Lessons:**

Our findings suggested that COL5A2 gene mutation (c1997G > A p.P659P) may be associated with cEDS but did not reveal other severe complications.

## 1 Introduction

Ehlers–Danlos syndrome (EDS) is a heritable connective tissue disorder characterized by affecting skin, joint, ligaments, vasculature and internal organs. The prevalence of EDS is estimated to be approximately 1/5000 births.^[1]^ EDS was first recognized 10 subtypes in the 1988 Berlin nosology.^[2]^ With the progress of biochemical and molecular basis, the Villefranche nosology was published in 1998 and this version recognized 6 subtypes, for which major and minor clinical criteria were defined.^[3]^ However, simply depending on clinical criteria was insufficient to distinguish several new EDS variants. Therefore, with the advent of next generation sequencing technique, the newly EDS classification is classified into 13 subtypes according to clinical manifestations and genetic mutations.^[4]^

Classical EDS (cEDS) is characterized by the presence of three major criteria: skin hyperextansibility, atrophic scarring and generalized joint hypermobility and 9 minor criteria such as bruising, doughy skin and family history.^[4]^ The minimal criteria for cEDS were skin hyperextensibility and atrophic scarring or generalized joint hypermobility, with and/or at least three minor criteria.^[4]^ Reduction of type V collagen is central to the pathogenesis of cEDS.^[5]^ Type V collagen is a fibrillar collagen which is involved in fibrillogenesis.^[6]^ Although previous studies confirmed that COL5A1 and COL5A2 are the major genetic mutation resulted in cEDS, the genotype-phenotype correlations of cEDS are still controversial. Hence, this study reported a case of cEDS with both clinical manifestations and COL5A2 gene mutation.

## 2 Case presentation

**Figure 1:**
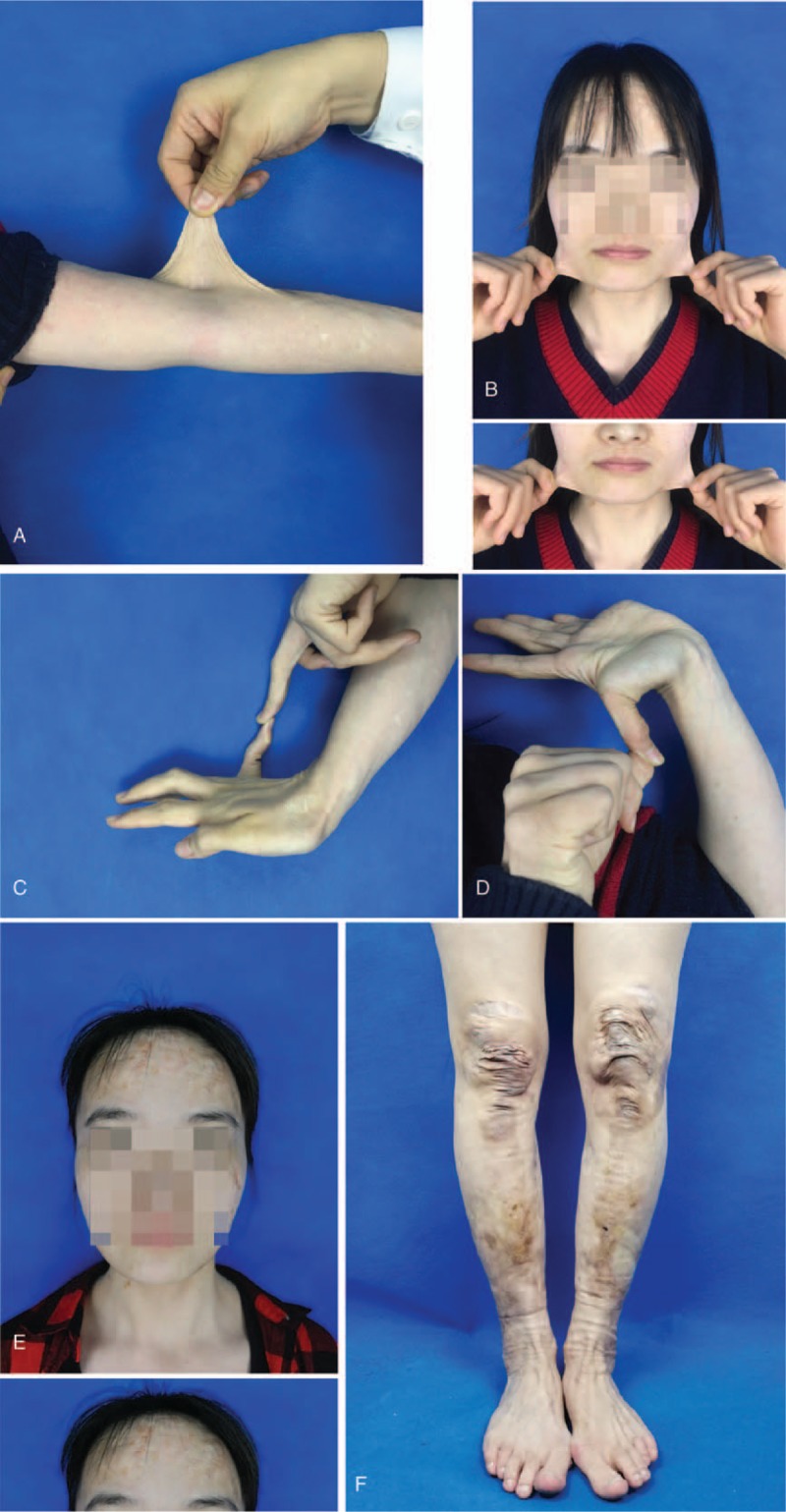
A and B. Hyperextensibility of elbow skin (A) and cheek skin (B); C and D. Joint hypermobility of fifth finger (C) and thumb (D); E and F. Papyraceous scar on forehead (E) and both knee and pretibial area (F).

**Figure 2:**
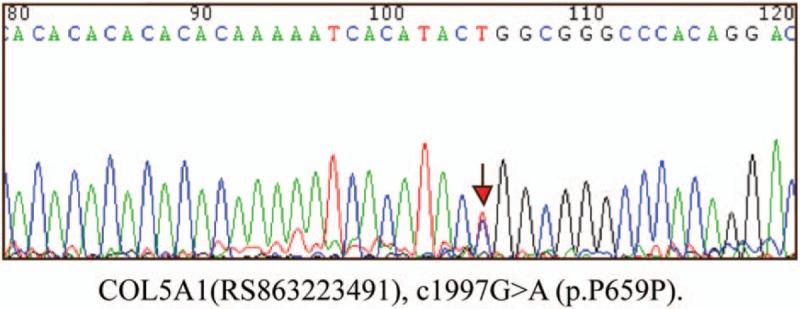
Genetic sequence analysis of *COL5A2* (c1997G > A, p. P659P) mutation in exon 2. The arrowhead denotes the position of the mutated base in the patient.

A 30-year-old female presented to the plastic-surgery clinic with widen atrophic scars on forehead, elbows, knees and pretibial area that had developed since childhood. Physical examination revealed skin hyperextensibility (Fig. 1A and B), joint hypermobility (Fig. 1C and D), papyraceous scar (Fig. 1E and F) and easy bruising. Ocular features such as epicanthal folds, infraorbital creases, and hypertelorism were also been observed in this patient. Results of laboratory tests and radiography examinations were normal. No vascular abnormalities were found, and the family history was negative. Whole exome sequencing revealed that the proband carried a heterozygous pathogenic variant, c1997G > A (p.P659P), in COL5A2 gene. Sanger sequencing of COL5A2 in this patient also confirmed the presence of this variant (Fig. 2). The diagnosis of the classical Ehlers–Danlos syndrome was made. The patient underwent facial scar resection and sutured in minimizing tension and perfect apposition to avoid the post-surgery scar formation. Follow-up 6 months after surgery, the wound remained a fine line scar. Patient has provided informed consent for publication of the case.

## 3 Discussion and conclusion

cEDS is a multisystem disorder which is unclear at the clinical and molecular level. In this study, we reported a patient with skin hyperextensibility, widened atrophic scars, generalized joint hypermobility and COL5A2 gene mutation. Skin hyperextensibility is the manifestation that skin can be stretched over 1.5 cm for the forearms and 3 cm for neck, elbow, and knee.^[3]^ Most patients have widened atrophic scar at forehead, elbow, knee and pretibial area and the scars can be seen in more than one body site. Joint hypermobility is evaluated by the Beighton score,^[3]^ and Beighton score >5 is considered positive for the presence of joint hypermobility. The elbow skin of our patient could be lifted over 6 cm, and with a Beighton score >5. Over 90% of EDS cases are classic and hypermobile EDS,^[7]^ and more than 90% of classical EDS patients harbor mutations in genes encoding type V collagen (*COL5A1* or *COL5A2*).^[8]^ Although about 80% patients have cEDS harbor mutation in *COL5A1*, *COL5A2* gene mutation results in severe end of cEDS spectrum.^[9]^ Previously, published study reported 2 patients with more severe cEDS due to a *COL5A2* mutation, (c.1977G > A) and (c.2499 + 2T > C), respectively.^[10]^ The patient harboring a *COL5A2* mutation (c.1997G > A) had several severe symptoms such as petus excavatum, scoliosis, hand and feet deformity, recurrently dislocations, and chronic pain. However, in this study, although whole exome sequencing revealed a same pathogenic variant, c1997G > A (p.P659P), in exon 2 of *COL5A2* gene, the patient only presented with 3 major diagnostic criteria: skin hyperextensibility, joint hypermobility, and widened atrophic scar without other complications. Therefore, genetic mutation might not be the only factor shaping the clinical manifestations.

Marfan syndrome, EDS, and Loeys–Dietz syndrome are examples of heritable connective tissue disorders that show some overlapping clinical manifestations.^[7]^ The diagnosis of Marfan syndrome is made based on the presence of cardiovascular features, aortic root dilatation, ectopia lentis and genetic screening of FBN1.^[11]^ On the other hand, Loeys–Dietz syndrome had no formal diagnostic criteria but the presence of aortic or dissection and the mutations of the TGFβ signaling pathway should be sufficient to make the diagnosis of Loeys–Dietz syndrome.^[12]^ Considerable phenotypic overlap is observed in the Marfan syndrome, Loeys–Dietz syndrome and EDS, which makes the clinical diagnosis uncertainly and highlights the crucial of molecular analysis confirmation.

Currently, there is no treatment which is developed to specifically eliminate cEDS. Only a series of preventive procedures are applicable, including closing dermal wounds in two layers with tension and avoiding repetitive heavy lifting.^[13]^ Cardiovascular diseases should be treated to avoid severe aortic root dilation and mitral valve prolapse, and follow-up echocardiogram should be performed once a year.^[8]^ Careful follow-up should be recommended for pregnancy and be aware of the risk of premature rupture of the membranes.

In conclusion, the presence of skin hyperextensibility, joint hypermobility and widened atrophic scar, combined with a pathogenic variant, c1997G > A (p.P659P), in exon 2 of *COL5A2* gene, may be a presenting sign of cEDS. More data are needed to determine the etiology of EDS so that an optimal treatment regimen can be produced for patients.

## Author contributions

**Supervision:** Tao Zan.

**Writing – original draft:** Xiangwen Xu.

**Writing – review & editing:** Zhichao Wang.

## References

[1] Vanakker O Callewaert B Malfait F The genetics of soft connective tissue disorders. Annu Rev Genomics Hum Genet 2015;16:229–55. 26002060 10.1146/annurev-genom-090314-050039

[2] Beighton P de Paepe A Danks D International nosology of heritable disorders of connective tissue, Berlin, 1986. Am J Med Genet 1988;29:581–94. 3287925 10.1002/ajmg.1320290316

[3] Beighton P De Paepe A Steinmann B Ehlers-Danlos syndromes: revised nosology, Villefranche, 1997. Am J Med Genet 1998;77:31–7. 9557891 10.1002/(sici)1096-8628(19980428)77:1<31::aid-ajmg8>3.0.co;2-o

[4] Malfait F Francomano C Byers P The 2017 international classification of the Ehlers–Danlos syndromes. Am J Med Genet Part C Semin Med Genet 2017;175:8–26. 28306229 10.1002/ajmg.c.31552

[5] De Paepe A Malfait F The Ehlers-Danlos syndrome, a disorder with many faces. Clin Genet 2012;82:1–1. 22353005 10.1111/j.1399-0004.2012.01858.x

[6] Malfait F De Paepe A Molecular genetics in classic Ehlers-Danlos syndrome. Am J Med Genet C Semin Med Genet 2005;null: 17-23. 10.1002/ajmg.c.30070 16278879

[7] Meester JAN Verstraeten A Schepers D Differences in manifestations of Marfan syndrome, Ehlers-Danlos syndrome, and Loeys-Dietz syndrome. Ann Cardiothorac Surg 2017;6:582–94. 29270370 10.21037/acs.2017.11.03 PMC5721110

[8] Bowen JM Sobey GJ Burrows NP Ehlers-Danlos syndrome, classical type. Am J Med Genet C Semin Med Genet 2017;175:27–39. 28192633 10.1002/ajmg.c.31548

[9] Symoens S Syx D Malfait F Comprehensive molecular analysis demonstrates type V collagen mutations in over 90% of patients with classic EDS and allows to refine diagnostic criteria. Hum Mutat 2012;33:1485–93. 22696272 10.1002/humu.22137

[10] Ritelli M Dordoni C Venturini M Clinical and molecular characterization of 40 patients with classic Ehlers-Danlos syndrome: identification of 18 COL5A1 and 2 COL5A2 novel mutations. Orphanet J Rare Dis 2013;8:58. 23587214 10.1186/1750-1172-8-58 PMC3653713

[11] Dietz HC Cutting GR Pyeritz RE Marfan syndrome caused by a recurrent de novo missense mutation in the fibrillin gene. Nature 1991;352:337–9. 1852208 10.1038/352337a0

[12] Van Hemelrijk C Renard M Loeys B The Loeys-Dietz syndrome: an update for the clinician. Curr Opin Cardiol 2010;25:546–51. 20838339 10.1097/HCO.0b013e32833f0220

[13] Malfait F Wenstrup RJ De Paepe A Clinical and genetic aspects of Ehlers-Danlos syndrome, classic type. Genet Med 2010;12:597–605. 20847697 10.1097/GIM.0b013e3181eed412

